# Application of mammography-based radiomics signature for preoperative prediction of triple-negative breast cancer

**DOI:** 10.1186/s12880-022-00875-6

**Published:** 2022-09-14

**Authors:** Shuai Ge, Yu Yixing, Dong Jia, Yang Ling

**Affiliations:** 1 The First Affiliated Hospital of Soochow University, Soochow, 215006 People’s Republic of China; 2 Suzhou Municipal Hospital, Soochow, 215006 People’s Republic of China

**Keywords:** Triple-negative breast cancer, Mammography, Radiomics

## Abstract

**Objective:**

This study is aimed to explore the value of mammography-based radiomics signature for preoperative prediction of triple-negative breast cancer (TNBC).

**Materials and methods:**

Initially, the clinical and X-ray data of patients (n = 319, age of 54 ± 14) with breast cancer (triple-negative—65, non-triple-negative—254) from the First Affiliated Hospital of Soochow University (n = 211, as a training set) and Suzhou Municipal Hospital (n = 108, as a verification set) from January 2018 to February 2021 are retrospectively analyzed. Comparing the mediolateral oblique (MLO) and cranial cauda (CC) mammography images, the mammography images with larger lesion areas are selected, and the image segmentation and radiomics feature extraction are then performed by the MaZda software. Further, the Fisher coefficients (Fisher), classification error probability combined average correlation coefficients (POE + ACC), and mutual information (MI) are used to select three sets of feature subsets. Moreover, the score of each patient’s radiomics signature (Radscore) is calculated. Finally, the receiver operating characteristic curve (ROC) is analyzed to calculate the AUC, accuracy, sensitivity, specificity, positive predictive value, and negative predictive value of TNBC.

**Results:**

A significant difference in the mammography manifestation between the triple-negative and the non-triple-negative groups (*P* < 0.001) is observed. The (POE + ACC)-NDA method showed the highest accuracy of 88.39%. The Radscore of triple-negative and non-triple-negative groups in the training set includes − 0.678 (− 1.292, 0.088) and − 2.536 (− 3.496, − 1.324), respectively, with a statistically significant difference (*Z* = − 6.314, *P* < 0.001). In contrast, the Radscore in the validation set includes − 0.750 (− 1.332, − 0.054) and − 2.223 (− 2.963, − 1.256), with a statistically significant difference (*Z* = − 4.669, *P* < 0.001). In the training set, the AUC, accuracy, sensitivity, specificity, positive predictive value and negative predictive value of TNBC include 0.821 (95% confidence interval 0.752–0.890), 74.4%, 82.5%, 72.5%, 41.2%, and 94.6%, respectively. In the validation set, the AUC, accuracy, sensitivity, specificity, positive predictive value and negative predictive value of TNBC are of 0.809 (95% confidence interval 0.711–0.907), 80.6%, 72.0%, 80.7%, 55.5%, and 93.1%, respectively.

**Conclusion:**

In summary, we firmly believe that this mammography-based radiomics signature could be useful in the preoperative prediction of TNBC due to its high value.

## Introduction

Due to its rapidly increasing incidence rate yearly, breast cancer has become the most predominant tumor disease in women globally [1]. Based on immunohistochemical characteristics, the dreadful breast cancer can be classified into triple-negative breast cancer (TNBC) and non-triple-negative breast cancer (NTNBC). The occurrence of TNBC is often distinguished by the lack of estrogen receptor (ER), progesterone receptor (PR) expression, and over-expression of human epidermal growth factor receptor 2 (HER-2) [2]. Although the occurrence rate accounts for only 10–20% [3], the TNBC subtype is the most aggressive and malignant, as well as the worst prognosis among all the breast cancers [4, 5]. Nonetheless, early prognosis and subsequent effective therapeutic strategies could improve the life expectancy of TNBC patients [6].

Due to the small size of tissue specimens and the heterogeneity of the tumors, the needle biopsy specimens are often preferred to assess immunohistochemistry in breast cancer [7]. However, this needle biopsy-based analysis is not ideal as it may not represent the entire tumor. To address this limitation, radiomics technology has emerged as an alternative due to its ability to convert medical images into high-dimensional data for quantitative research [8]. Recently, mammography and MRI radiomics have been applied in some practical applications. For instance, mammography imaging predicts the breast cancer axillary lymph nodes metastasis, and breast MRI radiomics predicts the response of breast cancer masses to chemotherapy. Among these approaches, mammography has become a routine screening method for middle-aged high-risk women due to its convenience, cost-effectiveness, and ability to clearly show calcification [9]. Motivated by these aspects, this study demonstrates the analysis of the mammography images of breast cancer, aiming to explore the application value of mammography-based radiomics signature in the preoperative prediction of TNBC.

## Materials and methods

### Clinical data

A retrospective analysis of the data was performed considering all female breast cancer patients who underwent mammography in the First Affiliated Hospital of Soochow University and Suzhou Municipal Hospital from January 2018 to February 2021. On the one hand, the inclusion criteria for selecting subjects were set as follows: 1. Women aged ≥ 18 years with newly diagnosed breast cancer patients with mammography images; 2. Histologically confirmed invasive breast cancer; and 3. Breast cancer with the pathological examination of ER, PR, and HER2 results [10]. On the other hand, the exclusion criteria were set as follows: 1. Patients with stage-IV (metastatic) breast cancer; 2. History of other malignant tumors; 3. Incomplete images of the two positions of the mammography images cannot be evaluated; and 4. Patients who have received neoadjuvant chemotherapy or endocrine therapy. According to the above criteria, a total of 319 female patients (TNBC = 65 and NTNBC = 254) aged from 25 to 83 years old, with an average age of 54 ± 14 years old, were enrolled. The patients from the First Affiliated Hospital of Soochow University were denoted as the training group (n = 211, 40 cases of TNBC, 171 cases of NTNBC), and patients from Suzhou Municipal Hospital were denoted as an external verification group (n = 108, 25 cases of TNBC, 83 cases of TNBC). Notably, patients (n = 319) were all non-special types of invasive breast cancer, in which several patients (n = 32) were with ductal carcinoma in-situ (composition of < 20%). With the declaration of the hospital ethics committee, the informed consent from these patients was exempted.

### Mammography image analysis

A digital mammography machine (Hologic Selenia, Hologic Medical Systems, Boston, USA) was used to capture images by scanning mediolateral oblique (MLO) and cranial-caudal (CC) positions of subjects in both hospitals. The mammography images are exported from the picture archiving and communication system (PACS) in the BPM format. All mammography images were assessed by two radiologists with more than 5 years of mammography diagnosis experience, discussed, and reached a consensus. The assessment was performed considering the following details: (1) The size of the lesion, *i.e.,* the largest measured diameter of the lesion on the image of the body position with larger lesions; (2) The location of the lesion, *i.e.,* the left breast and the right breast. (3) The Mammography manifestation, *i.e.,* refer to the 5^th^ edition breast imaging report and data system (breast imaging-reporting and data system, BI-RADS), including masses, calcifications, masses, and calcifications, structural distortions, as well as asymmetric densification.

### Radiomics analysis

**Fig. 1:**
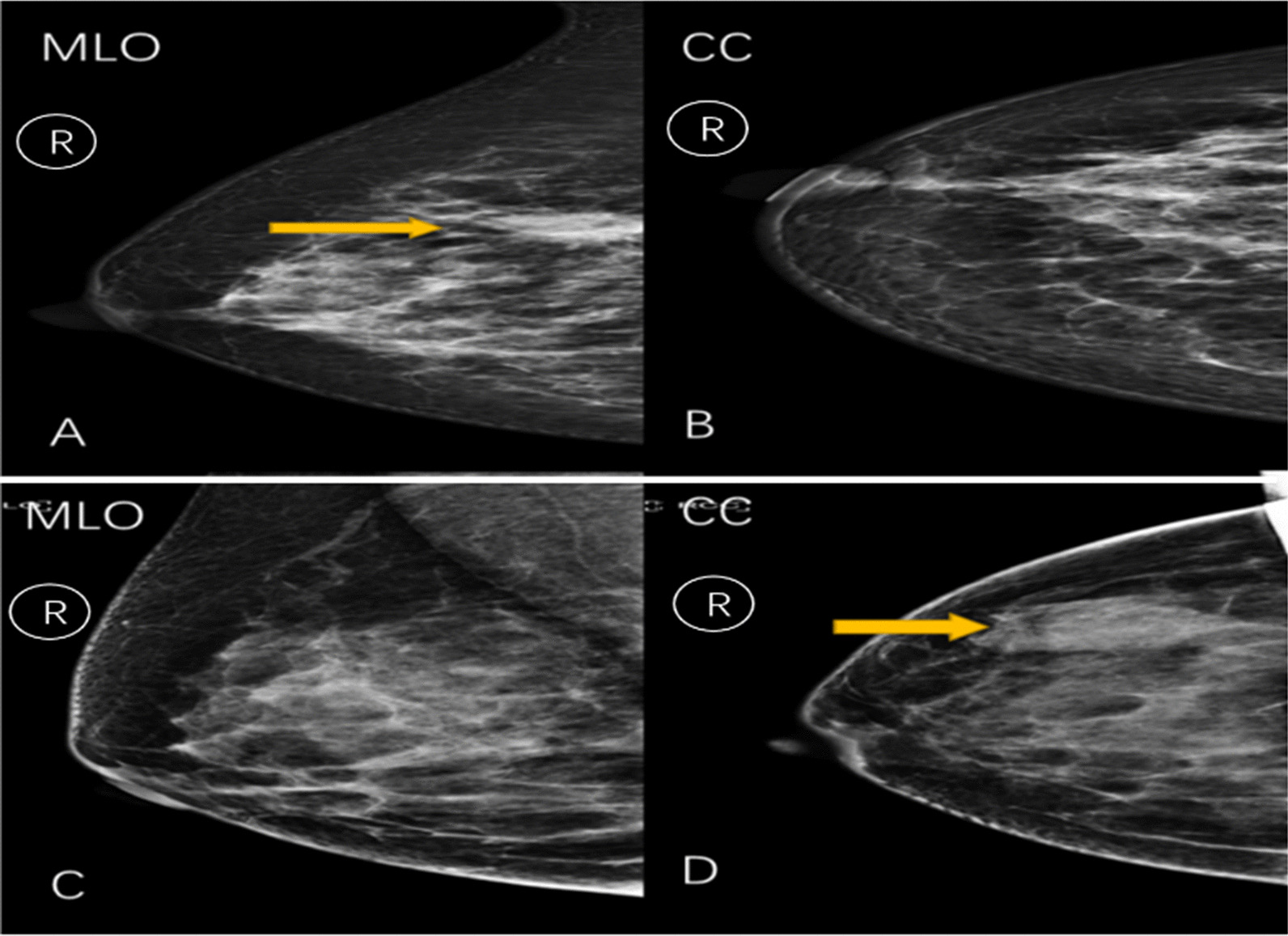
**A** and **B** The combination of surgical pathology, ultrasound, and MRI images of a patient (female, 48 years old) determines an irregular mass in the upper inner quadrant of the right breast. **A** A mass shadow over the right breast is seen in the MLO position in radiography. **B** No obvious lesion is seen in the CC position; Hence, we have chosen the radiomics features from the MLO. **C** and **D** The combination of surgical pathology, ultrasound, and MRI images of a patient (female, 65 years old) shows a well-defined mass in the upper outer quadrant of the right breast. **C** A mass shadow is seen in the MLO position in radiography. **D** No obvious lesion is seen in the CC position. **C** No obvious lesion is observed in the photographic MLO position. **D** A mass shadow is observed in the right lateral breast in the CC position. Hence, we have chosen the radiomics features from the CC

MaZda4.6 software (http://www.eletel.p.lodz.pl/mazda/) was employed to perform radiomics analysis on the training set of TNBC and NTNBC images. (1) Image uniformity processing: μ + 3σ (where μ is the average value of the image gray value and σ is the standard deviation of the gray image value) was selected in the MaZda software to perform the image gray uniformization processing and to minimize the contrast and the brightness of the image on the gray value of the image [11]. (2) Mammography image segmentation: As the lesions of some patients were clearly displayed on only one body position image, some cases could not be drawn simultaneously in two body positions. Therefore, this study compared the lesion size on the MLO and CC mammography images of the same patient and selected the body position image with a clear lesion and a larger area for segmentation (Fig. 1).

Among the subjects, 80 cases of breast cancer nodule images were segmented by physicians to evaluate the differences between and within the observers. (3) Radiomics quantitative feature extraction: MaZda software was used to extract quantitative features of mammography images, including gray-level histogram (GLH), absolute gradient (GRA), gray-level co-occurrence matrix (GLCM), gray-level run-length matrix (GLRLM), auto-regressive model (ARM), and wavelets transform (WAV). (4) Feature selection: The selection was performed using three feature selection methods provided by the MaZda software, such as Fisher coefficients (Fisher), classification error probability combined average correlation (POE + ACC), and the relative information measurement method (mutual information, MI). Further, the extracted quantitative features were screened, and 10 optimal feature parameters were automatically selected by the MaZda software to obtain 3 sets of feature subsets. The nonlinear discriminant analysis (NDA) provided by the B11 module of the MaZda software was employed to perform discriminant analysis on the 3 sets of feature subsets and calculated the accuracy of judging TNBC, respectively. (5) Standardization of raw data: The Z-Score algorithm in SPSS software was used to standardize the raw data of 10 characteristic parameters of all patients. (6) Construction of radiomics signature [12]: The standardized feature subset with the highest accuracy was further screened by the binary logistics regression. The linear fusion of selected features was used to construct the radiomics signature. Then, each patient’s Radscore was calculated, and the ROC curve analysis was performed to calculate the AUC, accuracy, sensitivity, specificity, positive predictive value, and negative predictive value of TNBC.

### Statistical methods

SPSS (v26.0) software was employed for statistical analysis. The qualitative data were expressed in terms of frequency. The quantitative data conforming to the normal distribution were expressed as mean ± standard deviation (S.D.). In contrast, the quantitative data that do not conform to the non-normal distribution were represented as the median (25% and 75% percentile). Independent-sample t-test (when in line with normal distribution) or Mann–Whitney U test (when not in line with normal distribution) was used to compare quantitative differences between the data of TNBC and NTNBC patients. A Chi-square test was used to analyze the location and X-ray manifestation of the lesion. The optimal feature subset was further screened by the binary logistic backward stepwise regression. Notably, the data were compared between groups, considering the *P* < 0.05 values statistically significant. The intraclass correlation coefficient (ICC) was used to analyze the consistency between two physicians manually segmenting the lesions and the same physician segmenting the lesions twice.

## Results

### Clinical and mammography data

According to the pathological results, the selected subjects were divided into 65 cases of TNBC patients and 254 cases of NTNBC patients. The average age of TNBC patients was ca. 53.83 ± 14.39 years old, and the average age of NTNBC patients was ca. 53.80 ± 14.42 years old, with no statistically significant difference (*P* = 0.989). To this end, the lesion sizes in TNBC and NTNBC patients were determined as 30.66 ± 17.63 mm and 31.12 ± 17.95 mm, with no statistically significant difference (*P* = 0.867). Among the 319 patients, the lesions were located on the different sides of the breast (161 cases in the right breast and 158 cases in the left breast). The mammography showed 98 cases of masses, 43 cases of calcification, 72 cases of masses plus calcification, 56 cases of structural distortion, and 50 cases of asymmetric compactness. Notably, no statistically significant difference was observed in age, lesion size, and lesion location between the training set and the validation set, TNBC and NTNBC groups of patients (*P* > 0.05). In contrast, the X-ray manifestations of TNBC and NTNBC patients were statistically significant (*P* < 0.001) (Tables 1, 2).

**Table 1:** Comparison of clinical indicators of breast cancer patients between the training and the verification groups

Group	Cases	Age	Size	Lesion location		X-ray manifestation					BI-RADS			
				Right	Left	Lumps	Calcification	Lumps and calcifications	Structural distortion	Asymmetric densification	4a	4b	4c	5
Training group	211	53.32 ± 14.03	32.10 ± 18.23	103	108	71	25	45	31	39	50	48	56	57
Verification group	108	54.75 ± 15.08	28.95 ± 17.01	58	50	27	18	27	25	11	31	23	25	29
*P* value		0.456	0.147	0.409		8.901					0.802			
Statistics		− 0.746a	− 1.452a	0.683b		0.064b					0.995			

Statistics: a: t value; b: X2

**Table 2:** Comparison of clinical indicators between patients with TNBC and NTNBC

Group	Cases	Age	Size	Lesion location		X-ray manifestation					BI-RADS			
				Right	Left	Lumps	Calcification	Lumps and calcifications	Structural distortion	Asymmetric densification	4a	4b	4c	5
TNBC	65	53.83 ± 14.39	30.66 ± 17.63	31	34	42	5	7	6	5	14	15	19	17
NTNBC	254	53.80 ± 14.42	31.12 ± 17.95	130	124	56	38	65	50	45	67	56	62	69
*P* value		0.989	0.867	0.616		< 0.001					0.778			
Statistics		− 0.014a	− 0.168a	0.252b		44.133b					1.097			

Statistics: a: t value; b: X2

### Diagnostic efficacy of radiomics signature for predicting TNBC

**Fig. 2:**
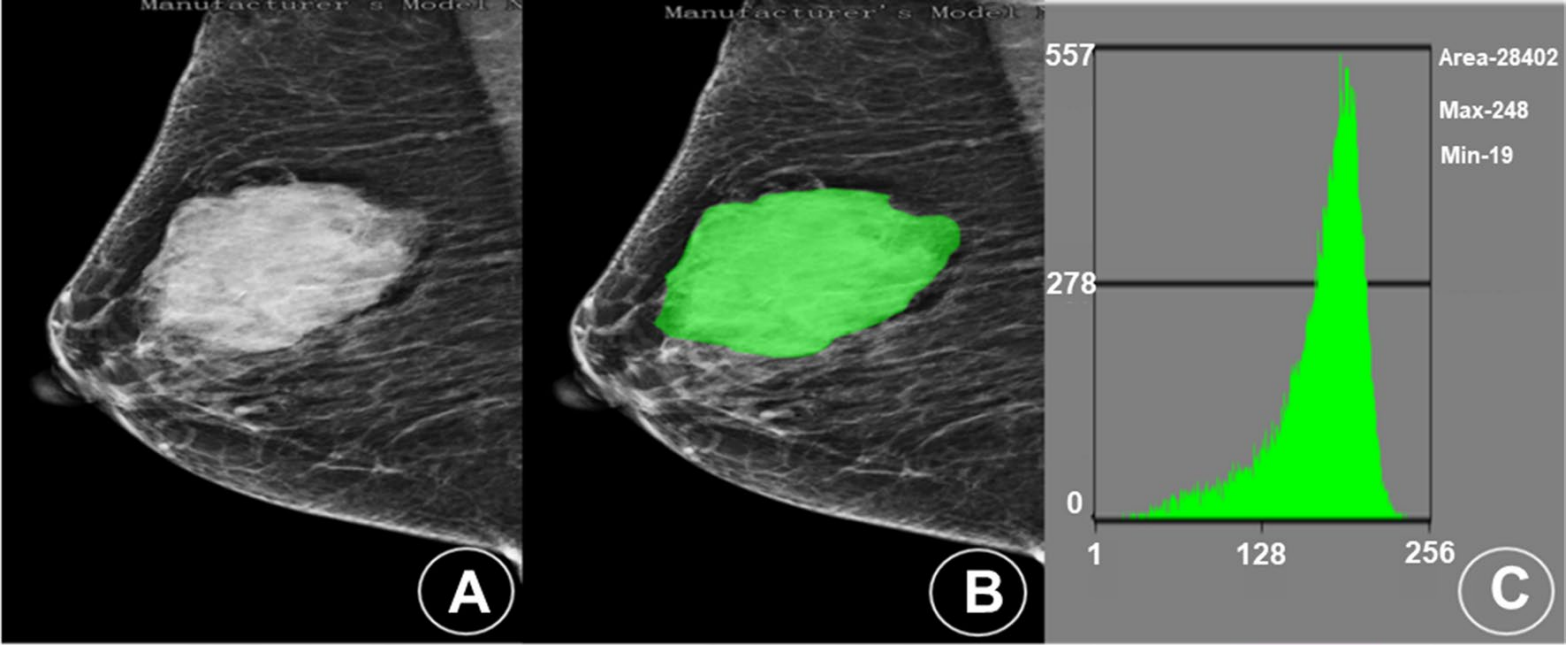
Mammogram and histogram analyses of a patient (female, 48 years old) with TNBC in the lateral quadrant of the left breast. **A** A mammography image shows an irregular nodule in the lateral quadrant of the left breast, with a length of about 7.9 cm and shallow lobes visible on the edge. **B** The MaZda image segmentation tool was applied to manually delineate the area of interest in the mammography and extract the radiomics features. **C** The gray level histogram shows the ROI in the lateral quadrant of the left breast

**Fig. 3:**
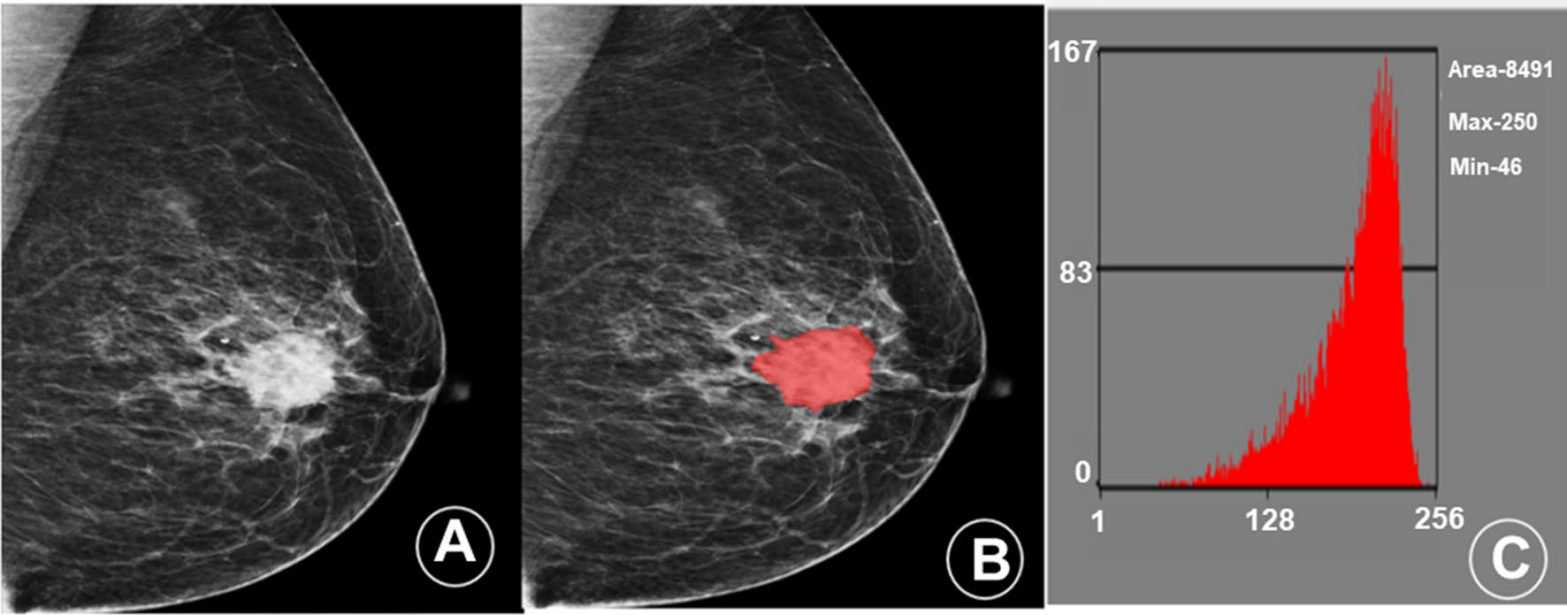
Mammogram and histogram of a patient (female, 52 years old) with NTNBC in the central area of the right breast. **A** A mammography image shows an irregular nodule in the central area of the right breast, with a length of about 3.4 cm, with lobes and burrs visible on the edge and small calcifications around it. **B** The MaZda image segmentation tool was applied to manually delineate the area of interest in the mammography and extract the radiomics features. **C** The gray level histogram shows the ROI in the central area of the right breast

Notably, the consistency in the manual segmentation of lesions between the two physicians was remarkable, with the intra-observer and inter-observer ICC values of 0.85 and 0.88, respectively. The MaZda software was employed to extract the quantitative features of the X-ray images of patients (a total of 343) in training set with TNBC (Fig. 2) and NTNBC (Fig. 3). To explore these aspects, three feature selection methods (Fisher, POE + ACC, and MI) were applied to screen the extracted quantitative features for predicting TNBC (Table 3). Further, the nonlinear discriminant analysis method was utilized to analyze the three feature subsets. It was observed from the results that the three feature subsets selected by Fisher (POE + ACC) and MI methods resulted in an accuracy of 84.52%, 88.39%, and 81.94% for predicting TNBC, respectively.

**Table 3:** Three subsets of feature methods of the training group predicting TNBC

Feature selection method	Parameter
Fisher	WavEnHH_s-3, WavEnLH_s-4, WavEnHL_s-4, WavEnHL_s-2, WavEnLH_s-3, GrMean, S(0,1) SumAverg, S(1,1) SumAverg, S(1,-1) SumAverg, S(2,0) SumAverg
POE + ACC	WavEnLH_s-4, Kurtosis, Perc.01%, Vertl_LngREmph, WavEnHH_s-5, Teta4, WavEnHL_s-5, 135dr_ShrtREmp, GrKurtosis, WavEnHH_s-1
MI	WavEnLL_s-2, WavEnLL_s-1, 135dr_Fraction, 135dr_LngREmph, WavEnLH_s-4, S(0,2) SumOfSqs, S(1,0) SumOfSqs, S(1,1) SumOfSqs, S(2,0) SumOfSqs, S(2,2) SumOfSqs

Fisher: Fisher parameter method; POE + ACC: Classification error rate combined average correlation coefficient method; MI: Related Information Measurement; Wavelet transform: WavEnHH_s-3,WavEnLH_s-4,WavEnHL_s-4, WavEnHL_s-2, WavEnLH_s-3, WavEnHH_s-5, WavEnHL_s-5, WavEnHH_s-1, WavEnLL_s-2, WavEnLL_s-1; Gradient model: GrMean, 135dr_ShrtREmp, GrKurtosis, 135dr_Fraction, 135dr_LngREmph; Gray Level Co-occurrence Matrix: S(0,1) SumAverg, S(1,1) SumAverg, S(1,-1) SumAverg, S(2,0) SumAverg, S(0,2) SumOfSqs, S(1,0) SumOfSqs, S(1,1) SumOfSqs, S(2,0) SumOfSqs, S(2,2) SumOfSqs; Histogram: Kurtosis, Perc.01%; Run matrix: Vertl_LngREmph; Autoregressive model: Teta4

**Fig. 4:**
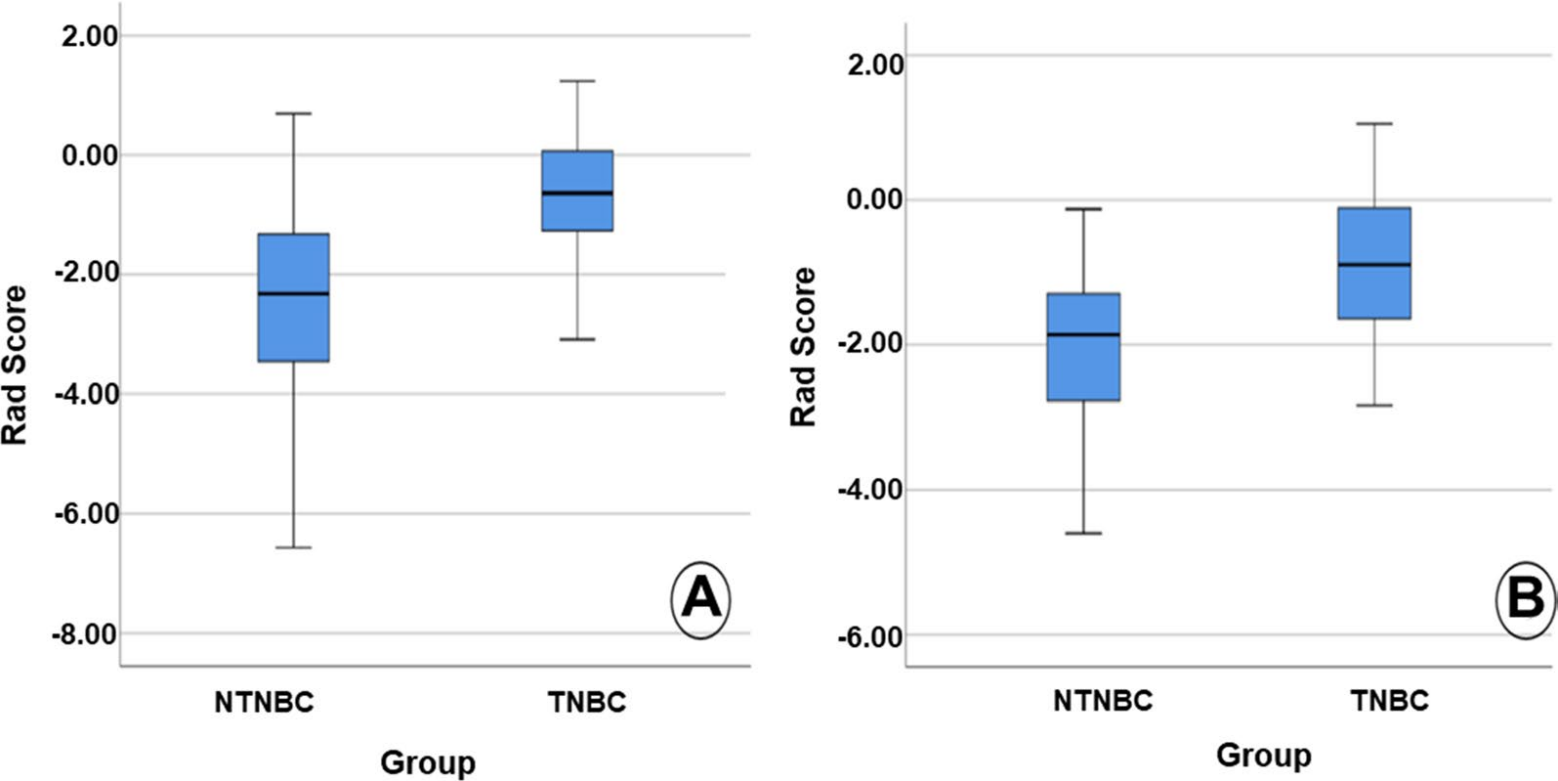
The box plot shows the Radscore of patients with TNBC and NTNBC in the training set (**A**) and the validation set (**B**)

**Fig. 5:**
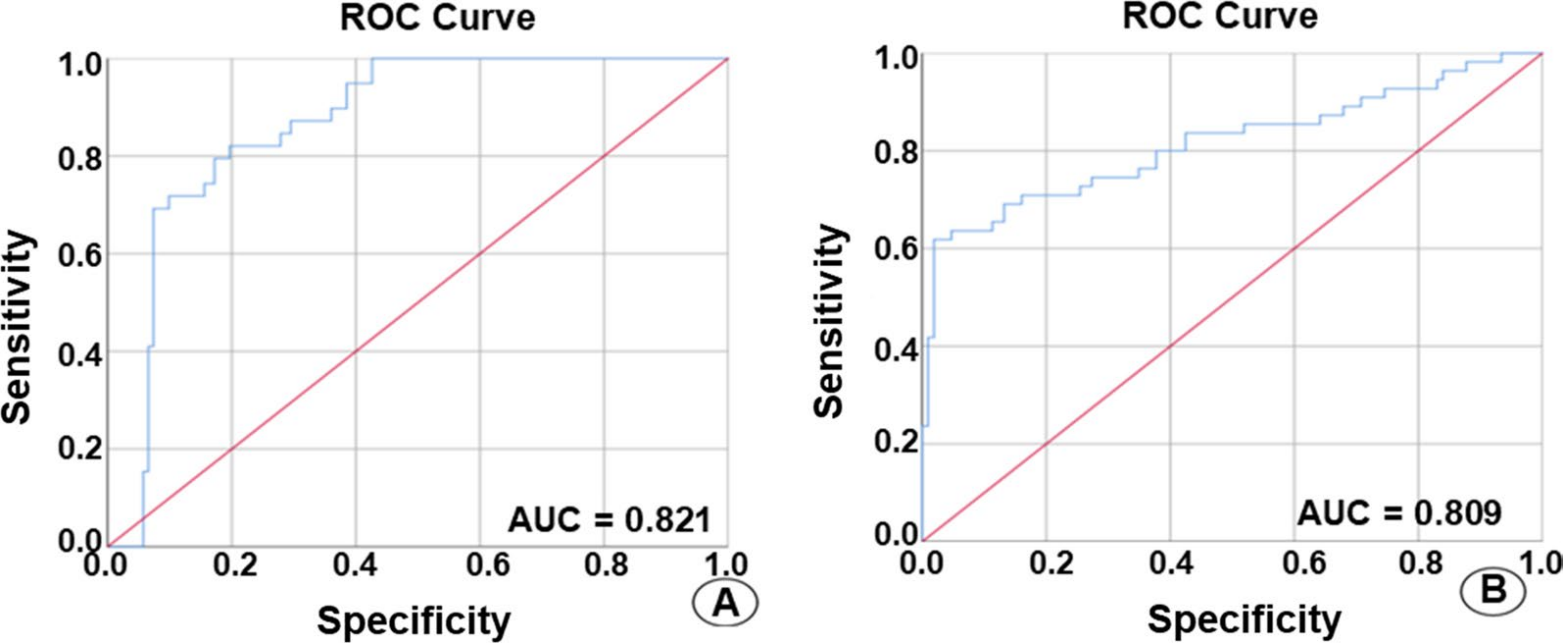
ROC curves illustrate the mammography radiomics signature prediction of the **A** training and **B** validation sets of TNBC

The Z-Score algorithm was used to standardize all the original feature data of the training and validation sets of the 10 features selected by the POE + ACC method with the highest accuracy. Further, the binary logistics regression was applied to screen the data. The linear fusion of standardized data selection features was then used to construct the radiomics signature: Radscore = − 0.664 × ZWavEnLH_s4 + 0.542 × ZKurtosis − 0.99 × ZPerc.01% − 0.813 × ZVertl_LngREmph − 1.738 × ZWavEnHH_s1 − 2.097. The Radscore of each patient was calculated. The Radscore of TNBC and NTNBC in the training set included − 0.678 (− 1.292, 0.088) and − 2.536 (− 3.496, − 1.324), respectively, with the statistically significant difference (Z = − 6.314, *P* < 0.001). Similarly, the Radscore of TNBC and NTNBC in the validation set resulted in − 0.750 (− 1.332, − 0.054) and − 2.223 (− 2.963, − 1.256), respectively, with a statistically significant difference (Z = − 4.669, *P* < 0.001) (Fig. 4). Then, the ROC curve analysis was performed using the obtained Radscore (Fig. 5). Further, the calculated AUC, accuracy, sensitivity, specificity, positive predictive value, and negative predictive value of TNBC in the predicted training set included 0.821 (95% acceptable confidence interval 0.752–0.890), 74.4%, 82.5%, 72.5%, 41.2%, and 94.6%, respectively. Whereas the AUC, accuracy, sensitivity, specificity, positive predictive value and negative predictive value of TNBC in the validation set included 0.809 (95% confidence interval 0.711–0.907), 80.6%, 72.0%, 80.7%, 55.5%, and 93.1%, respectively (Table 4).

**Table 4:** The efficacy of mammography radiomics signature in predicting TNBC

	AUC	95% Confidence interval	Accuracy (%)	Sensitivity (%)	Specificity (%)	Positive predictive value (%)	Negative predictive value (%)
Training group	0.821	0.752–0.890	74.4	82.5	72.5	41.2	94.6
Verification group	0.809	0.711–0.907	80.6	72.0	80.7	55.5	93.1

*AUC* area under the curve

## Discussion

This study is aimed to explore the implication of mammography radiomics value and predict TNBC in women before surgery. Accordingly, the experimental results showed that the radiomics signature possessed a certain value in determining the TNBC breast tumors, indicating a new indicator for preoperative diagnosis of TNBC. Radiomics is often preferred to extract the characteristic information of related lesions through images [13]. The extracted information describes the heterogeneity of tumors more intuitively and quantitatively to overcome the shortcomings of traditional diagnosis. In this study, five radiomics features were extracted, including two WAV features, two GLH features, and one GLRLM feature. Indeed, the WAV features were obtained by wavelet decomposition calculation of the intensity and texture features of the original image, which were focused on different frequency ranges within the tumor volume. To this end, the GLH features described the distribution of voxel intensities within the image region defined by the mask through commonly used and basic metrics. In addition, the GLRLM features quantified the gray level runs, defined as the length in the number of consecutive pixels with the same gray level value.

In recent years, the radiomics method has been employed to predict benign and malignant breast tumors, molecular typing, regional lymph node metastasis, and to explore the effect of neoadjuvant chemotherapy. Notably, TNBC patients possess a certain degree of resistance to endocrine therapy, indicating the non-availability of clinically effective treatment methods. Several reports indicated that the mammography manifestations of TNBC often presented features of benign lesions. Misdiagnosing such lesions as benign breast diseases might result in delayed treatment [14]. Therefore, accurately predicting TNBC before surgery is of great significance to the prognosis of the patient and the clinician to further formulate treatment methods for the patient.

Recently, the analysis of the imaging characteristics of TNBC has become a hot research topic in exploring its early prediction. Several research studies demonstrated that the image characteristics could be correlated to TNBC. In an instance, Yang et al. [15] observed that TNBC was more often presented as a mass than NTNBC in the X-ray findings, in which masses were mostly round, oval, or lobed, with clear borders and fewer irregularities. The morphological features of leaf-like or shallowly lobed at the edge could be due to some typical benign tumors, such as clear and smooth tumor edges, which were usually related to high-grade breast cancer with fast proliferation [16, 17]. The tumor cells proliferated faster and induced lesser stromal reactions, creating a clearer boundary between the tumor and surrounding normal tissues. In an instance, it was elucidated that TNBC and NTNBC patients possessed statistically significant differences in their X-ray findings (*P* < 0.001) and the linear manifestations of TNBC patients with masses (64.6%), which was in agreement with the above conclusion [17]. In another instance, Yang and colleagues [15] found that TNBC was less associated with microcalcification (15%), in which the proportions of HER2-positive and ER-positive patients with microcalcification were reported as 55% and 48%, respectively, which were in agreement with the results of this study. Notably, TNBC, as non-intraductal cancer, grows rapidly and develops to the invasive stage without prominent in-situ cancer components or precancerous lesions, indicating the lack of calcification.

Ma and colleagues [18] reported that the predictive value of the CC + MLO dual-view radiomics model was higher than that of the CC and MLO radiomics models alone. The selected radiomics contained all texture features, which were not verified by the validation set. However, this study adopted external verification, with higher repeatability and more objective and reliable results. It was observed that the results of the prediction model of this study were slightly higher than those of results reported by Zhang et al. The plausible reason behind the difference might be because of abundant extracted 324 radiomic features and the 3 feature selection methods provided by MaZda software to screen and then used logistic regression to obtain 5 independent predictive indicators, to construct radiomics signature in this study [19]. However, in Zhang et al.'s study, only 14 radiomics features were extracted, and independent predictive indicators were not used to construct radiomics signature. Moreover, they observed that the AUC of TNBC diagnosed based on the radiomics signature of conventional chest CT was lower than 0.76 in both the training set and the verification set, which was lower than the obtained results in this study [20]. Moreover, in the chest CT, the patient's supine position could not fully display the breast glands and lesions. Together, we believe that the mammography radiomics features provide certain practical application value in predicting TNBC.

## Data Availability

The datasets generated during this study are available from the corresponding author upon reasonable request.
